# Arctigenin: a lignan from *Arctium lappa*

**DOI:** 10.1107/S1600536808021752

**Published:** 2008-07-19

**Authors:** Haiyan Gao, Guanglei Li, Junhe Zhang, Jie Zeng

**Affiliations:** a School of Food Science, Henan Institute of Science and Technology, Xinxiang 453003, People’s Republic of China

## Abstract

The title compound {systematic name: (3*R*-*trans*)-4-[(3,4-dimethoxy­phen­yl)meth­yl]-3-[(4-hydr­oxy-3-methoxy­phen­yl)meth­yl]-4,5-dihydrofuran-2(3*H*)-one}, C_21_H_24_O_6_, has a dibenz­yl­butyrolactone skeleton. The two aromatic rings are inclined at a dihedral angle of 68.75 (7)° with respect to each other. The lactone ring adopts an envelope conformation. A series of O—H⋯O and C—H⋯O hydrogen bonds contribute to the stabilization of the crystal packing. The absolute configuration was assigned on the basis of the published literature.

## Related literature

For related literature, see: Awale *et al.* (2006 ▶). For a similar structure, see: Bruno-Colmenárez *et al.* (2007 ▶).
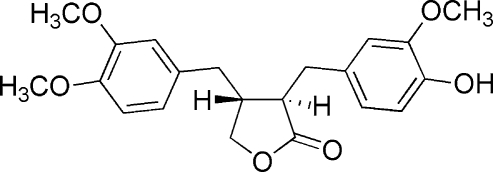


## Experimental

### 

#### Crystal data

C_21_H_24_O_6_
*M* *_r_* = 372.40
Orthorhombic, 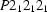

*a* = 9.4845 (19) Å
*b* = 10.065 (2) Å
*c* = 19.915 (4) Å
*V* = 1901.2 (7) Å^3^
*Z* = 4
Mo *K*α radiation
μ = 0.10 mm^−1^
*T* = 113 (2) K
0.14 × 0.12 × 0.10 mm

#### Data collection

Rigaku Saturn CCD area-detector diffractometer
Absorption correction: multi-scan (*CrystalClear*; Rigaku/MSC, 2005 ▶) *T*_min_ = 0.987, *T*_max_ = 0.991
13910 measured reflections
2581 independent reflections
2449 reflections with *I* > 2σ(*I*)
*R*_int_ = 0.037

#### Refinement

*R*[*F* ^2^ > 2σ(*F* ^2^)] = 0.032
*wR*(*F* ^2^) = 0.079
*S* = 1.06
2581 reflections
251 parameters
H atoms treated by a mixture of independent and constrained refinement
Δρ_max_ = 0.20 e Å^−3^
Δρ_min_ = −0.15 e Å^−3^

### 

Data collection: *CrystalClear* (Rigaku/MSC, 2005 ▶); cell refinement: *CrystalClear*; data reduction: *CrystalClear*; program(s) used to solve structure: *SHELXS97* (Sheldrick, 2008 ▶); program(s) used to refine structure: *SHELXL97* (Sheldrick, 2008 ▶); molecular graphics: *SHELXTL* (Sheldrick, 2008 ▶); software used to prepare material for publication: *CrystalStructure* (Rigaku/MSC, 2005 ▶).

## Supplementary Material

Crystal structure: contains datablocks global, I. DOI: 10.1107/S1600536808021752/bt2739sup1.cif

Structure factors: contains datablocks I. DOI: 10.1107/S1600536808021752/bt2739Isup2.hkl

Additional supplementary materials:  crystallographic information; 3D view; checkCIF report

## Figures and Tables

**Table 1:** Hydrogen-bond geometry (Å, °)

*D*—H⋯*A*	*D*—H	H⋯*A*	*D*⋯*A*	*D*—H⋯*A*
O5—H5⋯O2^i^	0.90 (2)	2.04 (2)	2.8280 (17)	146 (2)
O5—H5⋯O6	0.90 (2)	2.22 (2)	2.6799 (18)	111.7 (17)
O5—H5⋯O1^i^	0.90 (2)	2.58 (2)	3.2406 (18)	130.8 (16)
C3—H3⋯O5^ii^	0.95	2.34	3.278 (2)	168
C14—H14*A*⋯O4^iii^	0.99	2.86	3.687 (2)	142
C14—H14*B*⋯O5^iv^	0.99	2.42	3.373 (2)	162
C20—H20⋯O4^iii^	0.95	2.53	3.446 (2)	162

Symmetry codes: (i) 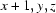; (ii) 
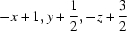
; (iii) 
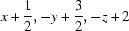
; (iv) 
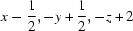
.

**Fig. 1.**
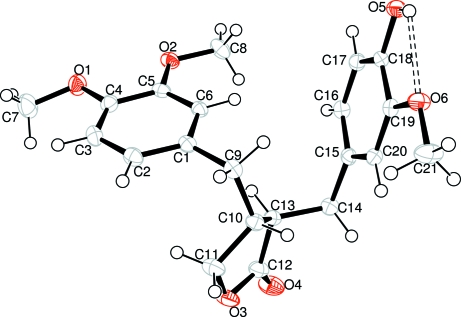
The molecular structure of (I) with the atom-numbering scheme and 50% probability displacement ellipsoids.

## supplementary crystallographic information

### Comment

Arctigenin has been identified as an antitumor agent with the ability to eliminate the tolerance of cancer cells to nutrient strarvation (Awale *et al.*,  2006).

The title compound  has a dibenzylbutyrolactone skeleton (Fig. 1). The two aromatic rings have a dihedral angle of 68.75 (7)°. The   lactone ring adopts an envelope conformation.   A series of O—H···O and C—H···O hydrogen bonds contribute to the stabilization of the crystal packing (Table 1).

### Experimental

Arctigenin was isolated from Chinese medicine Arctium lappa. Crystal blocks were obtained by natural evaporation of a methanolic solution.

### Refinement

In the absence of anomalous scatterers Friedel pairs were merged. The absolute configuration was set according to the literature (Awale *et al.*, 2006). The O-bound H atom was located in a difference map and freely refined. All other H atoms were positioned geometrically and refined as riding atoms, with U(H) = 1.2 U_eq_(CH and CH_2_) and C—H ranging from 0.95–1.0Å or U(H) = 1.5 U_eq_(CH_3_) and C_methyl_—H =0.99 Å. The methyl groups were allowed to rotate but not to tip.

### Crystal data

**Table:**

C_21_H_24_O_6_	*F*_000_ = 792
*M**_r_* = 372.40	*D*_x_ = 1.301 Mg m^−^^3^
Orthorhombic, *P*2_1_2_1_2_1_	Mo *K*α radiation λ = 0.71073 Å
Hall symbol: P 2ac 2ab	Cell parameters from 5995 reflections
*a* = 9.4845 (19) Å	θ = 2.3–27.9º
*b* = 10.065 (2) Å	µ = 0.10 mm^−^^1^
*c* = 19.915 (4) Å	*T* = 113 (2) K
*V* = 1901.2 (7) Å^3^	Block, colourless
*Z* = 4	0.14 × 0.12 × 0.10 mm

### Data collection

**Table:**

Rigaku Saturn CCD area-detector diffractometer	2581 independent reflections
Radiation source: rotating anode	2449 reflections with *I* > 2σ(*I*)
Monochromator: confocal	*R*_int_ = 0.037
Detector resolution: 7.31 pixels mm^-1^	θ_max_ = 27.8º
*T* = 113(2) K	θ_min_ = 2.1º
ω scans	*h* = −12→12
Absorption correction: multi-scan(CrystalClear; Rigaku/MSC, 2005)	*k* = −11→13
*T*_min_ = 0.987, *T*_max_ = 0.991	*l* = −26→16
13910 measured reflections	

### Refinement

**Table:**

Refinement on *F*^2^	Hydrogen site location: inferred from neighbouring sites
Least-squares matrix: full	H atoms treated by a mixture of independent and constrained refinement
*R*[*F*^2^ > 2σ(*F*^2^)] = 0.032	*w* = 1/[σ^2^(*F*_o_^2^) + (0.0475*P*)^2^ + 0.1518*P*] where *P* = (*F*_o_^2^ + 2*F*_c_^2^)/3
*wR*(*F*^2^) = 0.079	(Δ/σ)_max_ = 0.001
*S* = 1.06	Δρ_max_ = 0.20 e Å^−^^3^
2581 reflections	Δρ_min_ = −0.15 e Å^−^^3^
251 parameters	Extinction correction: SHELXL97 (Sheldrick, 2008), Fc^*^=kFc[1+0.001xFc^2^λ^3^/sin(2θ)]^-1/4^
Primary atom site location: structure-invariant direct methods	Extinction coefficient: 0.032 (3)
Secondary atom site location: difference Fourier map	

### Special details

**Table:**

Geometry. All e.s.d.'s (except the e.s.d. in the dihedral angle between two l.s. planes) are estimated using the full covariance matrix. The cell e.s.d.'s are taken into account individually in the estimation of e.s.d.'s in distances, angles and torsion angles; correlations between e.s.d.'s in cell parameters are only used when they are defined by crystal symmetry. An approximate (isotropic) treatment of cell e.s.d.'s is used for estimating e.s.d.'s involving l.s. planes.
Refinement. Refinement of *F*^2^ against ALL reflections. The weighted *R*-factor *wR* and goodness of fit *S* are based on *F*^2^, conventional *R*-factors *R* are based on *F*, with *F* set to zero for negative *F*^2^. The threshold expression of *F*^2^ > σ(*F*^2^) is used only for calculating *R*-factors(gt) *etc*. and is not relevant to the choice of reflections for refinement. *R*-factors based on *F*^2^ are statistically about twice as large as those based on *F*, and *R*- factors based on ALL data will be even larger.

### Fractional atomic coordinates and isotropic or equivalent isotropic displacement parameters (Å^2^)

**Table:**

	*x*	*y*	*z*	*U*_iso_*/*U*_eq_	
O1	0.07380 (13)	0.28900 (13)	0.73971 (6)	0.0287 (3)	
O2	0.23820 (12)	0.17843 (12)	0.82576 (5)	0.0228 (3)	
O3	0.38552 (14)	0.79457 (13)	0.92594 (6)	0.0329 (3)	
O4	0.34335 (15)	0.69713 (14)	1.02429 (6)	0.0362 (3)	
O5	0.96166 (13)	0.15373 (12)	0.87738 (6)	0.0226 (3)	
H5	1.036 (2)	0.195 (2)	0.8592 (10)	0.034*	
O6	1.04322 (12)	0.40766 (12)	0.88872 (6)	0.0241 (3)	
C1	0.44649 (17)	0.48348 (17)	0.79244 (7)	0.0196 (3)	
C2	0.35798 (18)	0.53926 (18)	0.74441 (8)	0.0230 (3)	
H2	0.3827	0.6219	0.7246	0.028*	
C3	0.23339 (18)	0.47622 (18)	0.72471 (8)	0.0247 (4)	
H3	0.1747	0.5159	0.6917	0.030*	
C4	0.19554 (17)	0.35649 (18)	0.75310 (8)	0.0226 (3)	
C5	0.28519 (17)	0.29699 (17)	0.80056 (7)	0.0196 (3)	
C6	0.40879 (17)	0.35955 (17)	0.81937 (7)	0.0192 (3)	
H6	0.4693	0.3180	0.8511	0.023*	
C7	−0.0319 (2)	0.3594 (2)	0.70329 (11)	0.0412 (5)	
H7A	−0.0003	0.3730	0.6569	0.062*	
H7B	−0.0485	0.4458	0.7246	0.062*	
H7C	−0.1196	0.3079	0.7033	0.062*	
C8	0.32263 (19)	0.11735 (19)	0.87673 (9)	0.0276 (4)	
H8A	0.3305	0.1774	0.9153	0.041*	
H8B	0.4168	0.0988	0.8588	0.041*	
H8C	0.2784	0.0340	0.8910	0.041*	
C9	0.57710 (16)	0.55340 (17)	0.81719 (7)	0.0209 (3)	
H9A	0.6165	0.6068	0.7799	0.025*	
H9B	0.6482	0.4855	0.8294	0.025*	
C10	0.55425 (17)	0.64553 (16)	0.87818 (8)	0.0213 (3)	
H10	0.6479	0.6827	0.8916	0.026*	
C11	0.4557 (2)	0.76166 (18)	0.86314 (9)	0.0287 (4)	
H11A	0.5100	0.8389	0.8464	0.034*	
H11B	0.3856	0.7361	0.8286	0.034*	
C12	0.39834 (18)	0.69247 (18)	0.96988 (8)	0.0257 (4)	
C13	0.48662 (16)	0.58224 (16)	0.94058 (8)	0.0192 (3)	
H13	0.4229	0.5086	0.9259	0.023*	
C14	0.58852 (17)	0.52865 (17)	0.99399 (7)	0.0211 (3)	
H14A	0.6435	0.6035	1.0129	0.025*	
H14B	0.5334	0.4884	1.0310	0.025*	
C15	0.68864 (17)	0.42650 (16)	0.96613 (8)	0.0194 (3)	
C16	0.65324 (16)	0.29296 (17)	0.96147 (7)	0.0205 (3)	
H16	0.5651	0.2633	0.9785	0.025*	
C17	0.74541 (16)	0.20166 (17)	0.93208 (7)	0.0206 (3)	
H17	0.7201	0.1105	0.9295	0.025*	
C18	0.87370 (16)	0.24419 (16)	0.90675 (7)	0.0188 (3)	
C19	0.91196 (16)	0.37751 (17)	0.91323 (7)	0.0187 (3)	
C20	0.82073 (16)	0.46753 (17)	0.94260 (7)	0.0192 (3)	
H20	0.8479	0.5580	0.9469	0.023*	
C21	1.08409 (19)	0.54337 (18)	0.89079 (10)	0.0329 (4)	
H21A	1.0905	0.5726	0.9376	0.049*	
H21B	1.0139	0.5973	0.8671	0.049*	
H21C	1.1761	0.5538	0.8691	0.049*	

### Atomic displacement parameters (Å^2^)

**Table:**

	*U*^11^	*U*^22^	*U*^33^	*U*^12^	*U*^13^	*U*^23^
O1	0.0263 (6)	0.0277 (7)	0.0322 (6)	−0.0037 (6)	−0.0070 (5)	0.0021 (5)
O2	0.0269 (6)	0.0178 (6)	0.0237 (5)	0.0014 (5)	0.0016 (5)	0.0025 (4)
O3	0.0430 (7)	0.0206 (6)	0.0353 (7)	0.0115 (6)	0.0054 (6)	0.0006 (5)
O4	0.0416 (7)	0.0322 (8)	0.0349 (7)	0.0107 (7)	0.0139 (6)	−0.0030 (6)
O5	0.0210 (5)	0.0180 (6)	0.0288 (6)	0.0004 (5)	0.0004 (5)	−0.0031 (5)
O6	0.0187 (5)	0.0194 (6)	0.0342 (6)	−0.0017 (5)	0.0050 (5)	−0.0041 (5)
C1	0.0205 (7)	0.0212 (8)	0.0171 (7)	0.0035 (7)	0.0033 (6)	0.0004 (6)
C2	0.0258 (8)	0.0209 (8)	0.0222 (8)	0.0017 (7)	0.0033 (6)	0.0036 (6)
C3	0.0247 (8)	0.0270 (9)	0.0222 (8)	0.0030 (7)	−0.0030 (6)	0.0041 (7)
C4	0.0225 (7)	0.0241 (9)	0.0214 (7)	0.0008 (7)	−0.0002 (6)	−0.0028 (6)
C5	0.0250 (8)	0.0159 (7)	0.0179 (7)	0.0034 (7)	0.0053 (6)	0.0007 (6)
C6	0.0218 (7)	0.0200 (8)	0.0158 (7)	0.0060 (7)	0.0023 (6)	−0.0002 (6)
C7	0.0310 (10)	0.0340 (11)	0.0584 (12)	−0.0034 (10)	−0.0192 (9)	0.0041 (10)
C8	0.0271 (8)	0.0259 (9)	0.0297 (9)	0.0047 (8)	0.0052 (7)	0.0108 (7)
C9	0.0193 (7)	0.0229 (8)	0.0206 (7)	0.0009 (7)	0.0030 (6)	0.0030 (6)
C10	0.0223 (7)	0.0177 (8)	0.0237 (7)	−0.0003 (7)	0.0015 (6)	0.0014 (6)
C11	0.0358 (9)	0.0193 (8)	0.0308 (9)	0.0049 (8)	0.0032 (7)	0.0029 (7)
C12	0.0250 (8)	0.0215 (8)	0.0307 (8)	0.0035 (7)	0.0022 (7)	−0.0025 (7)
C13	0.0187 (7)	0.0162 (8)	0.0227 (8)	0.0006 (6)	0.0017 (6)	−0.0015 (6)
C14	0.0213 (7)	0.0222 (8)	0.0198 (7)	0.0007 (7)	0.0028 (6)	0.0000 (6)
C15	0.0208 (7)	0.0223 (8)	0.0149 (7)	0.0024 (7)	−0.0018 (6)	0.0008 (6)
C16	0.0192 (7)	0.0220 (8)	0.0204 (7)	−0.0012 (7)	0.0001 (6)	0.0027 (6)
C17	0.0218 (7)	0.0182 (8)	0.0218 (7)	−0.0020 (7)	−0.0035 (6)	0.0019 (6)
C18	0.0193 (7)	0.0182 (8)	0.0190 (7)	0.0037 (6)	−0.0036 (6)	−0.0017 (6)
C19	0.0163 (7)	0.0217 (8)	0.0183 (7)	−0.0010 (6)	−0.0018 (6)	0.0009 (6)
C20	0.0204 (7)	0.0186 (8)	0.0188 (7)	−0.0003 (7)	−0.0023 (6)	−0.0009 (6)
C21	0.0260 (9)	0.0208 (9)	0.0519 (11)	−0.0064 (8)	0.0114 (8)	−0.0055 (8)

### Geometric parameters (Å, °)

**Table:**

O1—C4	1.366 (2)	C9—C10	1.543 (2)
O1—C7	1.426 (2)	C9—H9A	0.9900
O2—C5	1.369 (2)	C9—H9B	0.9900
O2—C8	1.432 (2)	C10—C11	1.526 (2)
O3—C12	1.355 (2)	C10—C13	1.537 (2)
O3—C11	1.455 (2)	C10—H10	1.0000
O4—C12	1.203 (2)	C11—H11A	0.9900
O5—C18	1.3665 (19)	C11—H11B	0.9900
O5—H5	0.90 (2)	C12—C13	1.507 (2)
O6—C19	1.3712 (19)	C13—C14	1.535 (2)
O6—C21	1.421 (2)	C13—H13	1.0000
C1—C2	1.391 (2)	C14—C15	1.506 (2)
C1—C6	1.404 (2)	C14—H14A	0.9900
C1—C9	1.508 (2)	C14—H14B	0.9900
C2—C3	1.398 (2)	C15—C16	1.389 (2)
C2—H2	0.9500	C15—C20	1.400 (2)
C3—C4	1.379 (2)	C16—C17	1.397 (2)
C3—H3	0.9500	C16—H16	0.9500
C4—C5	1.405 (2)	C17—C18	1.385 (2)
C5—C6	1.383 (2)	C17—H17	0.9500
C6—H6	0.9500	C18—C19	1.396 (2)
C7—H7A	0.9800	C19—C20	1.383 (2)
C7—H7B	0.9800	C20—H20	0.9500
C7—H7C	0.9800	C21—H21A	0.9800
C8—H8A	0.9800	C21—H21B	0.9800
C8—H8B	0.9800	C21—H21C	0.9800
C8—H8C	0.9800		
			
C4—O1—C7	116.50 (15)	C9—C10—H10	108.1
C5—O2—C8	116.88 (13)	O3—C11—C10	106.62 (13)
C12—O3—C11	109.96 (13)	O3—C11—H11A	110.4
C18—O5—H5	109.9 (15)	C10—C11—H11A	110.4
C19—O6—C21	116.75 (13)	O3—C11—H11B	110.4
C2—C1—C6	117.88 (16)	C10—C11—H11B	110.4
C2—C1—C9	122.16 (15)	H11A—C11—H11B	108.6
C6—C1—C9	119.94 (14)	O4—C12—O3	120.85 (17)
C1—C2—C3	121.34 (16)	O4—C12—C13	128.17 (17)
C1—C2—H2	119.3	O3—C12—C13	110.98 (13)
C3—C2—H2	119.3	C12—C13—C14	109.88 (13)
C4—C3—C2	120.12 (15)	C12—C13—C10	103.87 (13)
C4—C3—H3	119.9	C14—C13—C10	116.31 (13)
C2—C3—H3	119.9	C12—C13—H13	108.8
O1—C4—C3	125.08 (15)	C14—C13—H13	108.8
O1—C4—C5	115.51 (16)	C10—C13—H13	108.8
C3—C4—C5	119.40 (16)	C15—C14—C13	112.45 (12)
O2—C5—C6	125.01 (14)	C15—C14—H14A	109.1
O2—C5—C4	114.90 (15)	C13—C14—H14A	109.1
C6—C5—C4	120.07 (15)	C15—C14—H14B	109.1
C5—C6—C1	121.14 (15)	C13—C14—H14B	109.1
C5—C6—H6	119.4	H14A—C14—H14B	107.8
C1—C6—H6	119.4	C16—C15—C20	118.66 (15)
O1—C7—H7A	109.5	C16—C15—C14	122.23 (15)
O1—C7—H7B	109.5	C20—C15—C14	119.10 (15)
H7A—C7—H7B	109.5	C15—C16—C17	120.90 (15)
O1—C7—H7C	109.5	C15—C16—H16	119.6
H7A—C7—H7C	109.5	C17—C16—H16	119.6
H7B—C7—H7C	109.5	C18—C17—C16	119.94 (15)
O2—C8—H8A	109.5	C18—C17—H17	120.0
O2—C8—H8B	109.5	C16—C17—H17	120.0
H8A—C8—H8B	109.5	O5—C18—C17	119.10 (15)
O2—C8—H8C	109.5	O5—C18—C19	121.42 (15)
H8A—C8—H8C	109.5	C17—C18—C19	119.46 (15)
H8B—C8—H8C	109.5	O6—C19—C20	125.01 (15)
C1—C9—C10	114.99 (13)	O6—C19—C18	114.57 (14)
C1—C9—H9A	108.5	C20—C19—C18	120.42 (15)
C10—C9—H9A	108.5	C19—C20—C15	120.55 (15)
C1—C9—H9B	108.5	C19—C20—H20	119.7
C10—C9—H9B	108.5	C15—C20—H20	119.7
H9A—C9—H9B	107.5	O6—C21—H21A	109.5
C11—C10—C13	102.73 (13)	O6—C21—H21B	109.5
C11—C10—C9	113.07 (13)	H21A—C21—H21B	109.5
C13—C10—C9	116.48 (13)	O6—C21—H21C	109.5
C11—C10—H10	108.1	H21A—C21—H21C	109.5
C13—C10—H10	108.1	H21B—C21—H21C	109.5
			
C6—C1—C2—C3	1.7 (2)	O3—C12—C13—C14	137.62 (14)
C9—C1—C2—C3	−176.52 (14)	O4—C12—C13—C10	−167.32 (18)
C1—C2—C3—C4	0.2 (2)	O3—C12—C13—C10	12.54 (18)
C7—O1—C4—C3	−11.9 (2)	C11—C10—C13—C12	−21.56 (16)
C7—O1—C4—C5	167.03 (16)	C9—C10—C13—C12	−145.71 (14)
C2—C3—C4—O1	177.18 (15)	C11—C10—C13—C14	−142.41 (14)
C2—C3—C4—C5	−1.7 (2)	C9—C10—C13—C14	93.44 (17)
C8—O2—C5—C6	1.7 (2)	C12—C13—C14—C15	−174.92 (14)
C8—O2—C5—C4	−176.99 (13)	C10—C13—C14—C15	−57.32 (19)
O1—C4—C5—O2	1.0 (2)	C13—C14—C15—C16	−85.45 (19)
C3—C4—C5—O2	179.99 (14)	C13—C14—C15—C20	93.00 (17)
O1—C4—C5—C6	−177.79 (13)	C20—C15—C16—C17	−1.9 (2)
C3—C4—C5—C6	1.2 (2)	C14—C15—C16—C17	176.52 (14)
O2—C5—C6—C1	−177.86 (14)	C15—C16—C17—C18	−0.4 (2)
C4—C5—C6—C1	0.8 (2)	C16—C17—C18—O5	−179.14 (13)
C2—C1—C6—C5	−2.2 (2)	C16—C17—C18—C19	2.5 (2)
C9—C1—C6—C5	176.05 (13)	C21—O6—C19—C20	−3.5 (2)
C2—C1—C9—C10	89.17 (18)	C21—O6—C19—C18	176.67 (15)
C6—C1—C9—C10	−89.02 (18)	O5—C18—C19—O6	−0.7 (2)
C1—C9—C10—C11	−63.25 (19)	C17—C18—C19—O6	177.63 (13)
C1—C9—C10—C13	55.42 (19)	O5—C18—C19—C20	179.43 (14)
C12—O3—C11—C10	−17.30 (19)	C17—C18—C19—C20	−2.2 (2)
C13—C10—C11—O3	23.87 (17)	O6—C19—C20—C15	−179.96 (14)
C9—C10—C11—O3	150.24 (14)	C18—C19—C20—C15	−0.1 (2)
C11—O3—C12—O4	−177.32 (17)	C16—C15—C20—C19	2.2 (2)
C11—O3—C12—C13	2.8 (2)	C14—C15—C20—C19	−176.31 (14)
O4—C12—C13—C14	−42.2 (2)		

### Hydrogen-bond geometry (Å, °)

**Table:**

*D*—H···*A*	*D*—H	H···*A*	*D*···*A*	*D*—H···*A*
O5—H5···O2^i^	0.90 (2)	2.04 (2)	2.8280 (17)	146 (2)
O5—H5···O6	0.90 (2)	2.22 (2)	2.6799 (18)	111.7 (17)
O5—H5···O1^i^	0.90 (2)	2.58 (2)	3.2406 (18)	130.8 (16)
C3—H3···O5^ii^	0.95	2.34	3.278 (2)	168
C14—H14A···O4^iii^	0.99	2.86	3.687 (2)	142
C14—H14B···O5^iv^	0.99	2.42	3.373 (2)	162
C20—H20···O4^iii^	0.95	2.53	3.446 (2)	162

Symmetry codes: (i) *x*+1, *y*, *z*; (ii) −*x*+1, *y*+1/2, −*z*+3/2; (iii) *x*+1/2, −*y*+3/2, −*z*+2; (iv) *x*−1/2, −*y*+1/2, −*z*+2.

## References

[bb1] Awale, S., Lu, J., Kalaumi, S. K., Kurashima, Y., Tezuka, Y., Kadaota, S. & Esumi, H. (2006). *Cancer Res.* **66**, 1751–1757. 10.1158/0008-5472.CAN-05-3143 16452235

[bb2] Bruno-Colmenárez, J., Usubillaga, A., Khouri, N. & Díaz de Delgado, G. (2007). *Acta Cryst.* E**63**, o2046–o2047.

[bb3] Rigaku/MSC (2005). *CrystalClear* and *CrystalStructure* Rigaku Corporation, Tokyo, Japan.

[bb4] Sheldrick, G. M. (2008). *Acta Cryst.* A**64**, 112–122. 10.1107/S0108767307043930 18156677

